# Sex Differences in Behavioral Outcomes Following Temperature Modulation During Induced Neonatal Hypoxic Ischemic Injury in Rats

**DOI:** 10.3390/brainsci5020220

**Published:** 2015-05-22

**Authors:** Amanda L. Smith, Haley Garbus, Ted S. Rosenkrantz, Roslyn Holly Fitch

**Affiliations:** 1 Department of Psychology, University of Connecticut, 406 Babbidge Road, Unit 1020, Storrs, CT 06269, USA; E-Mails: haley.garbus@uconn.edu (H.G.); Roslyn.h.fitch@uconn.edu (R.H.F.); 2 Department of Pediatrics, University of Connecticut School of Medicine, 263 Farmington Avenue, Farmington, CT 06030, USA; E-Mail: rosenkrant@uchc.edu

**Keywords:** Temperature modulation, hypoxia ischemia, rodent model, rapid auditory processing, motor, learning/memory, preterm, neuroprotection, neurobehavioral, hypothermia

## Abstract

Neonatal hypoxia ischemia (HI; reduced oxygen and/or blood flow to the brain) can cause various degrees of tissue damage, as well as subsequent cognitive/behavioral deficits such as motor, learning/memory, and auditory impairments. These outcomes frequently result from cardiovascular and/or respiratory events observed in premature infants. Data suggests that there is a sex difference in HI outcome, with males being more adversely affected relative to comparably injured females. Brain/body temperature may play a role in modulating the severity of an HI insult, with hypothermia during an insult yielding more favorable anatomical and behavioral outcomes. The current study utilized a postnatal day (P) 7 rodent model of HI injury to assess the effect of temperature modulation during injury in each sex. We hypothesized that female P7 rats would benefit more from lowered body temperatures as compared to male P7 rats. We assessed all subjects on rota-rod, auditory discrimination, and spatial/non-spatial maze tasks. Our results revealed a significant benefit of temperature reduction in HI females as measured by most of the employed behavioral tasks. However, HI males benefitted from temperature reduction as measured on auditory and non-spatial tasks. Our data suggest that temperature reduction protects both sexes from the deleterious effects of HI injury, but task and sex specific patterns of relative efficacy are seen.

## 1. Introduction

Children born prematurely (<37 weeks gestation) or at very low birth weight (VLBW; <1500 grams) are at an increased risk for hypoxic ischemic brain injury (HI), with incidence rates rising as high as 60% at lower gestational ages/body weights (GA; [1]). HI refers to a reduction of blood and/or oxygen in the brain. In the preterm infant, HI events typically relate to the immaturity of the neurovascular system, and associated intraventricular/periventricular hemorrhage or non-hemorrhagic injuries that can lead to periventricular leukomalacia (PVL; [2,3]). In fact, most HI injuries among moderately to extremely preterm infants manifest as white matter damage, as well as some regionally specific volume reductions of gray matter in cortical and subcortical regions [3,4]. Though less common, HI can also occur in the late preterm infant (34–37 weeks gestational age), and damage patterns here are more similar to watershed and deep nuclei grey matter damage seen in term infants with HI [2,3,5,6,7]. This shift in patterns of injury reflects early vulnerability of oligodendrocyte progenitors, and subsequent vulnerability of grey matter (with the maturation of glutamatergic receptors).

In general, infants born prematurely tend to show extremely variable behavioral outcomes that are difficult to predict. For instance, preterm children might display subsequent impairments in motor abilities, with the most severe children being diagnosed with cerebral palsy (CP; [8,9,10]). Though the diagnosis of CP has fallen dramatically in recent years, mild and moderate motor impairments in children born prematurely are seen in about 30%–40% of cases [11,12,13,14,15]. Motor impairments may affect children’s ability to explore the environment and gain experiences, contributing to further cognitive delays and intellectual disabilities [16,17]. For example, infants with or at-risk for HI may show later deficits in rapid auditory processing (RAP; the ability to discriminate between rapidly changing auditory cues). These processing impairments may in turn be related to later language difficulties [18,19,20,21], including deficits in spelling and reading skills [22,23,24]. Language impairments are often accompanied by cognitive/memory impairments in the form of lower IQ scores, slow information processing speeds, and poor performance on spatial memory tasks [16,25,26,27,28].

Interestingly, most studies investigating the cognitive and behavioral outcomes of HI injury include only male subjects (animal studies), or a pooled combination of male and female preterm infants. This is surprising, given sporadic but compelling evidence that important differences in outcomes are seen between male and female preterms. For example, when males and females were matched for degree of HI damage, males were more adversely impacted, as evidenced by lower IQ scores [25,29,30,31,32,33], and other cognitive outcome indices [34,35,36,37]. Furthermore, being “male” has been identified as a universal risk factor for the incidence of neonatal stroke as well as developmental delays [37,38,39]. The underlying mechanism responsible for this sex difference in behavioral outcomes remains unknown, but theories involving hormones (*i.e.*, the possible debilitating effects of testosterone [40]), sex differences in cell death pathways [41,42,43,44], and genetics [45] have been proposed. However, it still remains unclear why sex differences in response to HI are seen. Yet this information is essential to the possible development and evaluation of neonatal neuroprotective strategies optimized to each sex.

Using animal models, we can further investigate the behavioral deficits mentioned above, and the question of why male and females display different behavioral outcomes following HI injury. Our lab and others have used the well-established Rice-Vannucci rat model of HI, where HI injury is experimentally induced on postnatal day (P) 7—roughly equating to injury in the late-preterm infant (34–36 gestational weeks (GW); [46,47,48,49,50]). Using this model, our lab has demonstrated behavioral deficits in male P7 HI rats, as assessed by tests of auditory processing, motor ability, spatial/non-spatial learning, working memory, and visual attention [25,51,52,53,54,55,56,57,58,59,60]. Our findings and those of others nicely parallel what is seen in the clinical population, thereby validating the use of the P7 HI model to study the consequences of neonatal HI. Furthermore, though studies are scarce, the handful of rodent studies looking at male and females separately consistently show a female advantage over males following neonatal/perinatal brain injury on various behavioral tasks [25,56,57,61,62].

In addition to investigating sex differences in behavioral outcomes, the P7 HI rodent model provides an ideal platform to explore possible therapeutic interventions for an HI injury. For example, body temperatures at/around the time of HI injury in infants have been found to play a particularly important role in modulating severity of outcomes [63]. Specifically, lower body temperatures (which have been shown to be extremely similar to reported brain temperature at the time [64]) lead to improved outcomes after neonatal HI insult. In fact, very small reductions in temperature (as little as 1 °C) are associated with prominent neuroprotection during the neonatal period [65,66]. Clinically, it has been found that temperature reduction during or shortly after an HI insult provides optimal neuroprotection, because lower temperature during the primary phase of HI (when there is a profound reduction in oxygen availability and metabolism, leading to depletion of high-energy metabolites and excessive depolarization of cells) taps a “window of opportunity” for amelioration [63]. In turn, this can lead to reduced morbidity and neurological impairments as children age [67,68]. Though hypothermia has primarily been used (and is considered standard practice) in term infants with hypoxic-ischemic encephalopathy (HIE), including head or whole body cooling, late-preterm infants who are at a high risk for HI events might also benefit from reduced body temperatures (though the practical application of hypothermia in the late-preterm infant may differ from term infants). Studies investigating hypothermia in infants at this age are scarce but do support further testing in pre-clinical animal models. For example, a preterm fetal sheep model has demonstrated that hypothermia following induced HI is associated with an overall reduction in hypoxia-induced loss of immature oligodendrocytes, as well as reduction in energy expenditure [69]. Benefits of lower body temperatures have also been substantiated in other animal studies, showing for example that cooling during an ischemic insult is associated with more favorable long-term outcomes [70,71,72]. However, benefits are bounded by evidence that extremely low body temperatures (approximately 5 °C lower than normal) also lead to a higher incidence of cell death in a piglet model [73]. Finally, and most important to the current study, animal studies have shown that females benefit more from reduced body temperatures than males following induced HI injury [74,75]. This sex difference could relate to cooling effects on caspase-3 activity, which is highly implicated in the caspase-dependent cell death pathway primarily activated by females [74,76].

While the above clinical and animal research studies suggest sex differences in behavioral outcomes following an HI insult—and also allude to the importance of temperature modulation—the literature directly assessing this complex topic (combined sex and temperature effects on HI) is scant. Therefore, the current study sought to explore the relationship between temperature and severity of HI insult in male and female P7 HI rats. We examined the performance of both sexes on a rota-rod task, rapid auditory processing (RAP) tasks, and maze tasks indexing spatial and non-spatial learning. Given prior evidence of a female HI advantage on most of these behavioral tasks, we hypothesized that female P7 HI rats would benefit more from lower body temperatures during HI insult as compared to P7 HI males. The findings presented here reinforce the importance of temperature modulation during and immediately after an HI insult. Our data support evidence on the neuroprotective effect of lower body temperatures, but also reveal whether lower temperatures have the same benefits for each sex.

## 2. Experimental Section

### 2.1. Subjects

Subjects were male (*n* = 32) and female (*n* = 32) Wistar rats born to time-mated dams (Charles River Laboratories, Wilmington, MA, USA) shipped to the University of Connecticut Bousfield vivarium on embryonic (E) day 5. Dams were housed in individual cages on a 12-h light/dark cycle and pups were born on approximately E22. On P1, pups were culled to litters of 5 females and 5 males. On P21, pups were weaned and pair-housed with like-treated like-sex littermates. Animals were single-housed when they reached adulthood. The UConn Care and Use Committee approved all procedures.

#### Induction of Hypoxia Ischemia

On P7, pups were randomly selected for HI or sham procedure, and assigned to the hypothermic or normothermic group. Groups were as follows: HI normothermic (*n* = 10 per sex), HI hypothermic (*n* = 10 per sex), and shams (*n* = 6 normothermic and 6 hypothermic per sex, subsequently pooled to *n* = 12 per sex). Pups were anesthetized with isoflourane (2.5%) and an incision was made vertically on the neck. For HI animals, the right common carotid artery was separated from surrounding tissue, and cauterized to restrict blood flow to the right cerebral hemisphere. The incision was sutured, and footpad injections were made for identification. Sham animals received a similar surgical procedure without artery cauterization. Following surgery, all normothermic pups (HI and sham) were placed in a temperature-controlled incubator with a warming lamp, while hypothermic pups (HI and sham) were placed in an unheated holding container with a warming lamp. Specifically, during hypoxia, HI normothermic pups were placed in an airtight container and subjected to 8% oxygen (balanced with nitrogen) for 120 min. The container was positioned atop a temperature-controlled slide warmer and with a warming lamp (to maintain nest temperatures; container air temperature averaged 36.1 °C). HI hypothermic pups were placed in an airtight container (also subjected to 8% oxygen balanced with nitrogen for 120 min), with only a warming lamp (container air temperature averaged 32.2 °C throughout exposure). Sham animals were placed in similar containers, following similar temperature controls. Body temperatures were recorded before and after hypoxia for all animals using a VeraTemp non-contact thermometer (Brooklands), which has been shown in our lab to yield temperatures highly consistent with rectal probe measures across varied environments (always relative +1.5 C°) in rats pups. The average nest temperature of all animals (taken prior to hypoxia) was 36.6 °C. Temperatures following hypoxia were as follows (reported as mean temperature ± standard error): male HI normothermic: 37.04 °C ± 0.49; male HI hypothermic: 35.76 °C ± 0.47; female HI normothermic: 37.29 °C ± 0.47; female HI hypothermic: 35.65 °C ± 0.47; male sham: 37.55 °C ± 0.43; female sham: 37.38 °C ± 0.43 (Table 1). The modest temperature decrease in our “hypothermic” condition was selected conservatively (*i.e.*, less than typically used in HIE hypothermic intervention), based on a dearth of information regarding whether cooling may have deleterious or beneficial effects in late preterm infants with HI events (and associated models). Following the implementation of this mild hypothermia, pups were returned to nest temperature before being placed back with the dam.

**Table 1:** Temperatures were taken before and after hypoxia to ensure that the average temperature of hypothermic animals was significantly lower than the temperature of normothermic animals. A 2 (Time) × 2 (Sex) × 3 (Treatment) repeated measures ANOVA revealed an overall Treatment effect (*p* < 0.001) and Time × Treatment interaction (*p* < 0.001). Tukey *post hoc* tests revealed significant differences after hypoxia between male HI hypothermic animals and male shams (*p* < 0.001) and between female HI hypothermic animals and female shams (*p* < 0.05), indicating that both male and female hypothermic groups had significantly lower temperatures than their sham counterparts. A uni-variate ANOVA assessing temperatures of *all* animals (HI and sham) post-hypoxia revealed a significant Temperature effect (*p* < 0.001), further confirming lower body temperatures in all hypothermic animals (regardless of Treatment and Sex).

Treatment Groups	Temperature Before Hypoxia (°C; mean ± SE)	Temperature After Hypoxia (°C; mean ± SE)
Male HI normothermic (*n* = 9)	36.61 ± 0.28	37.04°C ± 0.49
Male HI hypothermic (*n* = 10)	36.69 ± 0.26	35.76°C ± 0.47
Male Sham (pooled; *n* = 12)	36.79 ± 0.24	37.55°C ± 0.43
Female HI normothermic (*n* = 10)	36.42 ± 0.26	37.29°C± 0.47
Female HI hypothermic (*n* = 10)	36.53 ± 0.26	35.98°C ± 0.47
Female Sham (pooled; *n* = 12)	36.76 ± 0.24	37.38°C ± 0.43

Normal Nest Temperature: 36°C–38°C.

### 2.2. Behavioral Testing

One female HI normothermic animal was removed from the study due to seizure activity. Therefore, sixty-three animals were tested on all behavioral paradigms.

#### 2.2.1. Sensorimotor Task: Rota-Rod (P30–P32)

A rota-rod task was used to assess motor coordination and learning in the juvenile period. Animals were placed on a rotating rod that gradually accelerated from 4 rotations per minute (rpm) to 44 rpm, over the course of 5 min. Animals were given two trials a day for 3 days, and latency to fall off the rod was recorded per trial (in seconds). The average latency per day was used for analysis.

#### 2.2.2. Auditory Discrimination: Startle Reduction Paradigm

The startle reduction paradigm utilizes each animal’s acoustic startle response (ASR)—A large motor response to a startle-eliciting stimulus (SES; 105 dB white noise burst). When the SES is preceded by a pre-stimulus cue, pre-pulse inhibition (PPI; a reduction in the ASR) is observed, with the magnitude of attenuation indicating the degree of an animal’s detection of the cue (see [77] for review). This provides a measure of cue detectability, based on a comparison of ASRs following the pre-pulse cue (*i.e.*, cued *vs.* uncued trials). Where multiple cues were used in a session, mean values for each cue were compared to the mean uncued response. From these comparisons, attenuation (ATT) scores were calculated by dividing each animal’s cued trial score by their uncued trial score, and multiplying that score by 100 to get a percentage (cued trials/uncued trials * 100).

#### 2.2.3. Auditory Discrimination: Behavioral Testing Apparatus

Animals were placed on a Med Associated PHM-252B load cell platform in a black polypropylene cage. Output voltages from each platform were sent from a PHM-250-60 linear load cell amplifier to a Biopac MP100A-CE Acquisition system connected to a Power Macintosh G3. This system recorded the amplitude of each subject’s ASR in millivolts (mV) 150 ms from the onset of the SES (acquired as a waveform, using the program Acqknowledge). Each subject’s peak value served as the subject’s response amplitude for a given trial (in mV). Auditory stimuli were generated on a Pentium III Dell PC with custom programmed software and a Tucker Davis Technologies (RP2) real time processor, and amplified by a Niles SI-1260 Systems Integration Amplifier. Sound files were delivered through 10 Cambridge Soundworks MC100 loudspeakers placed 53 centimeters (cm) above the platforms.

#### 2.2.4. Auditory Discrimination: Normal Single Tone (NST; P32)

This task consisted of 103 cued or uncued trials presented in random order. Animals were tested for one session. Uncued trials consisted of a silent background followed by the SES, while cued trials included presentation of a 75-dB, 7-ms, 2300-Hz tone 50-ms prior to the SES. On cued trials, the cue-to-burst intervals varied (25, 50, 75, and 100 ms). ATT scores acted as a measure of cue detectability, with scores closer to 100% indicating poorer performance. This task provided a baseline measure to reveal any hearing or PPI deficits that could affect further testing.

#### 2.2.5. Auditory Discrimination: Silent Gap (SG) Procedure (0–100; P33–P37)

The silent gap detection task consisted of 299 trials per session, with one testing session a day for 5 consecutive days, in the juvenile period. On cued trials, a silent gap (ranging from 2 to 100 ms, embedded in the 75 dB broadband white noise) acted as the cue (50 ms prior to the SES). On uncued trials, the SES was presented randomly within the 75 dB broadband white noise (variable inter-trial interval (ITI)).

#### 2.2.6. Learning/Memory Assessments: Water Maze and Water Escape

Water maze tasks utilized a Sony Handcam DCR-TRV280 camera mounted above a 48-in. diameter tub filled with room-temperature water (~22 °C). The camera was used to record each subject’s path in the tub. A Smart Video Tracking System Version 2.5 connected to a Dell Dimension E521 computer translated each path, and recorded latency to find a submerged 8-in. diameter platform (2 cm below the surface).

Initially, animals were tested on a water escape task. This task provides an index of basic swimming ability, and acclimation to further water maze testing. Animals were placed in an oval tub (40.5 in × 21.5 in) filled with water, on the side opposite to a visible platform located at one end. Animals were required to swim to the visible platform. If an animal did not reach the platform after 45 s, it was guided there by an experimenter (10 s). All animals were able to complete this task regardless of experimental condition.

#### 2.2.7. Learning/Memory Assessments: Morris Water Maze (MWM; P73–P76)

In this task, animals are required to use spatial cues to locate a submerged platform that remains in a fixed location. To locate the platform, animals use extra-maze cues such as large black shapes painted on the surrounding walls and doorframe. No intra-maze cues are provided for this task. To begin, animals were placed in the maze at one of the four starting locations (N, S, E, or W quadrant of the pool). Animals were required to swim until finding the hidden submerged platform, which was always located in the southeast quadrant of the pool. On a given day, the start location varied randomly between trials, and the same start location was never used on a testing day. Each animal had 45 s to find the platform. If 45 s elapsed before the animal found the platform, the experimenter guided the rat to the platform and allowed the animal to sit and survey the room for 10 s. Each animal received 4 trials a day for 4 consecutive days, with the order of the starting quadrants changing each day.

#### 2.2.8. Learning/Memory Assessments: Non-Spatial Water Maze (P80–P83)

For this task, the testing apparatus is the same as the previously outlined MWM task (*i.e.*, a uniform circular black tub). However, here animals are required to use *intra-maze* cues to locate a submerged goal platform that is located in various quadrants of the pool. Intra-maze cues consisted of black and white vertical lines, horizontal lines, diagonal lines sloping down to the left, and diagonal lines sloping down to the right. These cues were placed in the four quadrants of the pool, and were changed at the beginning of each test trial. For each trial, the escape platform was located in a different quadrant of the pool (NW, NE, SW, SE), but was always associated with the vertical black and white lines cue (throughout testing). The starting location of the animal remained constant. For each trial, animals were given 45 s to locate and mount the platform before they were guided to the location and allowed to sit and survey the room for 10 s. Each animal received 4 trials a day for 4 consecutive testing days.

### 2.3. Statistical Analyses

All statistical analyses were performed using SPSS 15.0 software, alpha criterion 0.05 (IBM, Armonk, NY, USA). Two tailed analyses were used unless otherwise stated. Initial data analyses confirmed comparability of hypo- and normothermic sham data, which were therefore pooled into male and female sham groups (3 Treatments × 2 Sexes = 6 groups). For temperature analyses, an overall repeated measures analysis of variance (ANOVA) was performed for the 6 Treatment groups, as well as additional one-way ANOVAs to compare temperatures after hypoxia. Additional repeated measures ANOVAs were used to assess performance on behavioral tasks, with variables including Sex, Treatment (3 levels; sham, HI hypothermic, HI normothermic), Latency (for the rota-rod and maze tasks), Day, and Gap (for SG 0–100). Repeated measures ANOVAs were also performed for each task for males and females separately, followed by Tukey post-hoc tests. All behavioral graphs are presented with mean and standard error.

## 3. Results

### 3.1. Temperature Analysis

A 2 (Time) × 2 (Sex) × 3 (Treatment) repeated measures ANOVA revealed an overall Treatment effect (F(2, 57) = 15.561, *p* < 0.001), and a Time × Treatment interaction (F(2, 57) = 10.233, *p* < 0.001), reflecting a consistency in temperatures across groups before hypoxia, but a divergence after hypoxia (hypothermic lower; see Table 1). Further, overall Tukey *post hoc* tests revealed significant differences between male HI hypothermic animals and male shams (*p* < 0.001), and between female HI hypothermic animals and female shams (*p* < 0.005), indicating that we successfully lowered the body temperatures of hypothermic animals. Similar differences were seen for normothermic *versus* hypothermic HI animals after hypoxia, again indicating lower body temperatures in hypothermic HI animals overall. Additionally, a uni-variate ANOVA to assess the temperature of *all* hypothermic and normothermic animals post-hypoxia revealed a significant Temperature effect (F(1, 61) = 22.813, *p* < 0.001), indicating that regardless of Sex and Treatment, hypothermic animals displayed significantly lower body temperatures. Mean temperatures of all normothermic groups (HI, and pooled sham males and females) remained in the range of optimal nest temperature at all times (36 °C–38 °C; [78]).

### 3.2. Rota-Rod

**Figure 1:**
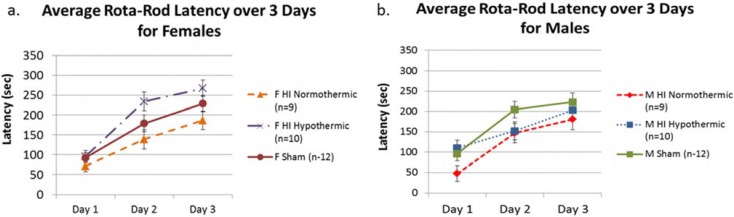
(**a**) A 3 (Day) × 3 (Treatment) repeated measures ANOVA revealed significantly different performances between female Treatment groups (*p* < 0.05). Further Tukey *post hoc* analyses revealed significantly better performance by female hypothermic animals compared to female HI normothermic animals (*p* < 0.05); (**b**) A 3 (Day) × 3 (Treatment) repeated measures ANOVA did not reveal any significant difference in performances for male Treatment groups, nor did further Tukey *post hoc* analyses.

A 3 (Day) × 2 (Sex) × 3 (Treatment) repeated measures ANOVA revealed an overall Treatment effect (F(2, 56) = 5.067, *p* < 0.05), indicating differences in performance between all Treatment groups. Based on *a priori* hypotheses, we performed 3 (Day) × 3 (Treatment) repeated measures ANOVAs within each sex separately. For females, this analysis revealed a significant overall Treatment effect (F(2, 28) = 4.484, *p* < 0.05), with a Tukey *post hoc* test revealing significant differences between female HI normothermic animals and hypothermic animals (*p* < 0.05; better performance in female hypothermic HI animals, confirming the protective effect of hypothermia; see Figure 1a). For males, a similar repeated measures ANOVA and Tukey *post hoc* test did not reveal any significant effects, indicating hypothermia did not benefit HI males on rota-rod performance (see Figure 1b).

### 3.3. NST

A 2 (Sex) × 3 (Treatment) uni-variate ANOVA did not reveal any significant differences in performance by Treatment group (F(2, 56) = 1.334, *p* > 0.05), or Sex (F(1, 56) = 0.104, *p* > 0.05), indicating all animals could hear, and display pre-pulse inhibition, as required for subsequent auditory testing.

### 3.4. SG 0–100

**Figure 2:**
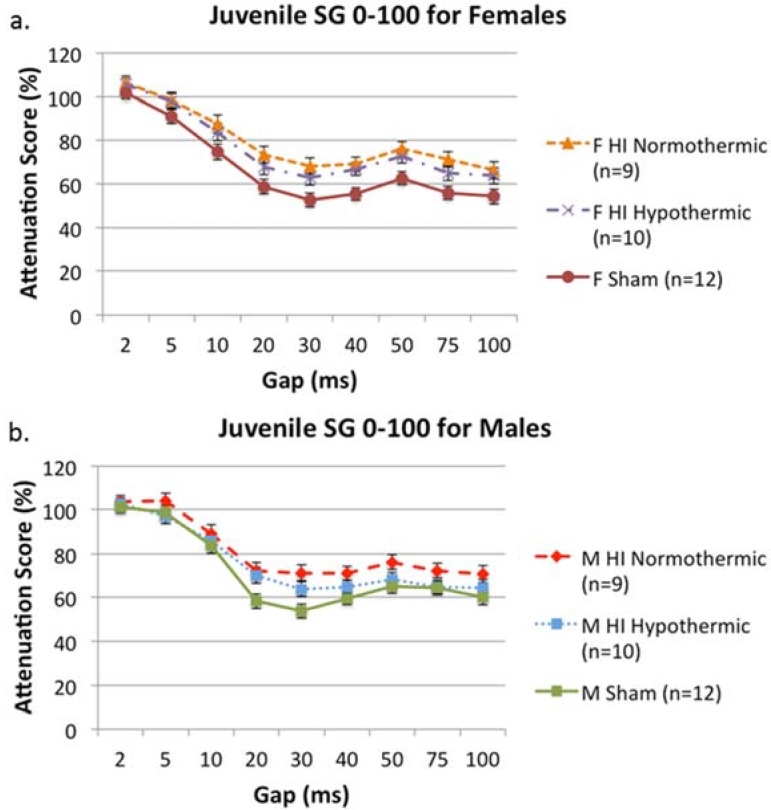
(**a**) A 5 (Day) × 9 (Gap) × 3 (Treatment) repeated measures ANOVA revealed significant overall differences between Treatment groups (*p* < 0.05). Further Tukey *post hoc* analyses revealed both HI normothermic animals and HI hypothermic animals performed significantly worse than shams (*p* < 0.05, *p* = 0.05, respectively); (**b**) A 5 (Day) × 9 (Gap) × 3 (Treatment) repeated measures ANOVA revealed significant overall differences between Treatment groups (*p* < 0.05). Further Tukey *post hoc* analyses revealed similar performances between HI hypothermic animals and shams (*p* > 0.05), but HI normothermic animals performed significantly worse than shams (*p* < 0.05).

An overall 5 (Day) × 9 (Gap) × 2 (Sex) × 3 (Treatment) repeated measures ANOVA revealed an effect of Treatment, indicating different performance between groups (F(2, 56) = 10.522, *p* < 0.001). Based on *a priori* hypotheses, we performed additional 5 (Day) × 9 (Gap) × 3 (Treatment) repeated measures ANOVAs for each sex separately, to assess whether hypothermia had a similar effect in each sex. For females, this analysis revealed a significant Treatment effect (F(2, 28) = 4.740, *p* < 0.05), and a Tukey *post hoc* test revealed significant differences between female HI normothermic animals and female shams (*p* < 0.05; shams better), as well as differences between female HI hypothermic animals and shams (indicating both HI groups, regardless of temperature, performed significantly worse than shams on a silent gap detection task; see Figure 2a). For males, a 5 (Day) × 9 (Gap) × 3 (Treatment) repeated measures ANOVA revealed an overall effect of Treatment (F(2, 28) = 4.241, *p* < 0.05), and a Tukey *post hoc* test only revealed a significant difference between male HI normothermic animals and male shams. This result indicates that HI normothermic animals performed worse than shams, while HI hypothermic animals performed comparably to shams (confirming the beneficial effect of lower body temperatures on behavior (*p* < 0.05; see Figure 2b)).

### 3.5. MWM

**Figure 3:**
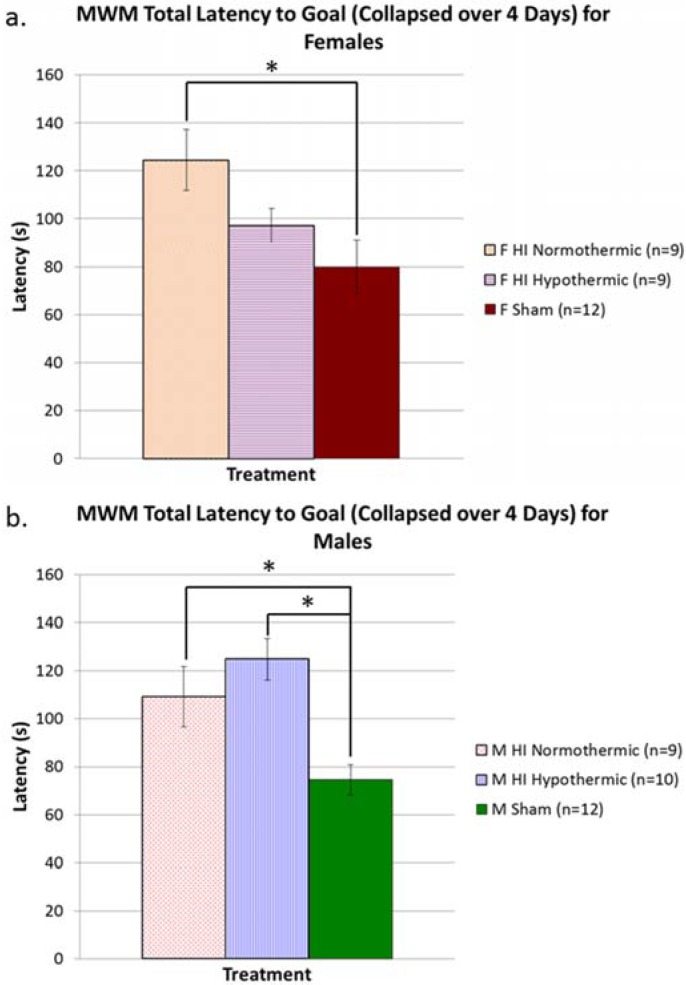
(**a**) A one-way ANOVA to assess Treatment effects revealed significant differences between Treatment groups (*p* < 0.05). Further Tukey *post hoc* analyses revealed that HI normothermic animals performed significantly worse than shams (* *p* < 0.05), but HI hypothermic animals performed similarly to shams; (**b**) A one-way ANOVA to assess Treatment effects revealed significant differences between Treatment groups (*p* < 0.05). Further Tukey *post hoc* analyses revealed that both HI normothermic animals and HI hypothermic animals performed significantly worse than shams (* *p* < 0.05, * *p* < 0.005, respectively).

An overall ANOVA revealed a significant Treatment effect (F(2, 56) = 15.356, *p* < 0.001), as well as Treatment x Sex interaction (F(2, 56) = 6.613, *p* < 0.05), indicating different performances by Treatment groups, modulated by Sex. Individual one-way ANOVAs for each Sex were also performed, revealing a significant Treatment effect for both females (F(2, 28) = 4.548, *p* < 0.05), and males (F(2, 28) = 8.727, *p* < 0.05) (see Figure 3a,b). These results indicate that HI animals of both sexes performed worse than their sham counterparts. Further Tukey *post hoc* tests for females revealed a significant difference between female HI normothermic animals and female shams (*p* < 0.05; shams better). A Tukey *post hoc* test for males confirmed a significant difference between male HI normothermic animals and male shams (*p* < 0.05; shams better), and also between male HI hypothermic animals and male shams (*p* < 0.005; shams better).

### 3.6. Non-Spatial Maze

**Figure 4:**
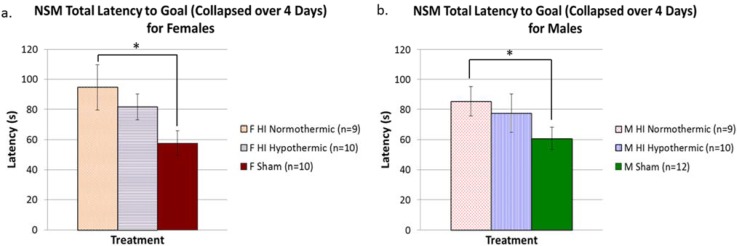
(**a**) A one-way ANOVA to assess Treatment effects did not reveal any overall significant differences in the performance of each group. However, individual t-tests revealed that HI normothermic animals performed significantly worse than shams (* *p* < 0.05) while HI hypothermic animals performed similar to shams (confirming the beneficial effect of hypothermia in ameliorating behavioral deficits); (**b**) A one-way ANOVA to assess Treatment effects did not reveal any overall significant differences in the performance of each group. However, individual t-tests revealed that HI normothermic animals performed worse than shams (* *p* < 0.05), while HI hypothermic animals performed similarly to shams (confirming the beneficial effect of hypothermia in ameliorating behavioral deficits).

An overall ANOVA revealed a nearly significant Treatment effect (F(5, 54) = 1.927, *p* = 0.1). Based on *a priori* hypotheses, we performed separate one-way ANOVAs for males and females (see Figure 4a,b) to assess Treatment effects in both sexes separately. For females, we found a nearly significant Treatment effect (F(2, 28) = 2.724, *p* = 0.08), and individual *t*-tests between each group revealed a significant difference between female HI normothermic animals and female shams (*t*(19) = 2.109, *p* < 0.05; shams better). For males, a one-way ANOVA did not reveal any significant differences, but individual t-tests between groups (based on *a priori* hypotheses) revealed a significant difference between male HI normothermic animals and male shams (*t*(19) = 2.033, *p* = 0.05; shams better).

## 4. Discussion

The current study sought to examine the effect of lower body temperatures during hypoxia/ ischemia in male and female P7 HI injured rats, and particularly to determine whether both sexes responded similarly. We have previously used the P7 HI model to reveal behavioral deficits in both sexes, and have found that females appear to exhibit some innate protection from the deleterious behavioral effect of induced hypoxia ischemia [25]. Here, we investigate whether an active neuroprotective intervention interacts with sex, and particularly whether females benefit more from lower body temperatures than males (as measured by behavioral outcomes). Results from the current study did show an overall benefit from hypothermia in both of HI hypothermic groups (both male and female), as has also been shown in other studies explicitly investigating the effects of active cooling on HI outcomes [74,79,80,81]. We attribute behavioral differences in our HI groups to differences in body temperature, with evidence of protection in both sexes from lower body temperatures. However, females seemed to benefit *more* from lower temperature than males, with some task-specific differences observed.

Initially, we found that HI females benefitted more from lower body temperatures as compared to HI males on a rota-rod task assessing motor ability and motor learning. This finding supported our initial hypothesis. That is, female HI hypothermic animals performed significantly better than female HI normothermic, while male HI animals displayed poor performance regardless of temperature (see Figure 1). It is important to note that these results were seen with testing over multiple days on the rota-rod task, and Treatment differences would not have been apparent (especially in females; see Figure 1a) if only one day of testing was administered. By using multiple testing days, data reveal that female HI hypothermic animals actually had the highest rate of motor learning (see Figure 1a)—likely reflecting protection from a lower body temperature during injury. Our findings are also consistent with general sensorimotor evidence from P7 HI males, where deficits are seen on these tasks (e.g., rota-rod [60,82]). It is possible that HI deficits on the rota-rod might be too severe in P7 HI males to be ameliorated, while more subtle female HI deficits could be attenuated. Though there are very few studies looking at the effects of temperature modulation in each sex following experimentally induced HI, our findings are consistent with the few studies on this topic (and these primarily used sensorimotor measures). For example, in an animal study by Fan *et al.*, [74] experimenters looked explicitly at active cooling (three hours post-insult), and found that females benefitted more from lower temperatures than males as measured by a sensorimotor task. Another study also showed that females demonstrated better *long-term* sensorimotor outcomes (assessed in adulthood) as compared to males following an HI injury when treated with hypothermia [75].

In contrast to these findings, on the silent gap task, males seemed to benefit more from hypothermia than females (see Figure 2b). This result was surprising, given evidence from a previous study revealing a slight innate HI female advantage on an FM sweep task (another task that assesses RAP ability; [25]). We do know that P7 HI males can be protected from rapid auditory processing deficits associated with an HI insult using other forms of neuroprotection (e.g., erythropoietin; [54,58]). Though the protective effects of erythropoietin were not investigated in P7 HI females, the results of McClure *et al.*, [54] and Alexander *et al.*, [58], coupled with the auditory results in the current study, might suggest that auditory deficits in males have more potential for amelioration than in females.

Finally, our maze tasks yielded contradictory results, with HI females showing a significant benefit of lower body temperature on a MWM task, but HI animals of both sexes benefitting equally from lower temperature on the non-spatial maze task (see Figure 3 and Figure 4). The protective effect seen in females on the MWM task parallels findings from another study conducted in our lab, wherein females were found to show an innate significant advantage in learning/memory outcomes following P7 HI [25]. Specifically, because we recently modified our HI procedure to employ more consistent nest temperatures throughout hypoxia (as shown here with “normothermic” HI effects), our prior evidence of a female HI advantage in outcomes probably reflects at least some protection in HI females from unintended cooling [25]. In other animal studies looking at rehabilitation training as a neuroprotectant, females were also found to display beneficial results on a spatial maze task, though males did not seem to be affected by any rehabilitation training [83]. However, the uniform benefit from hypothermia on the non-spatial maze for both sexes is a novel finding. For HI males, deficits on learning/memory tasks are not surprising, since they have been shown in previous studies [55,58,84,85,86,87,88]. Our results offer new evidence that these deficits can be ameliorated in both sexes with temperature modulation. The few animal studies examining the beneficial effect of hypothermia have shown amelioration of learning deficits in either pooled groups (male and female HI rats) or using only male HI animals (see [89] for review; [90]).

Overall, cooling during ischemia has also been shown to substantially decrease infarct volume and (when employed at optimal temperature), can lead to total protection in the brain [70,71,72,91]. Our current findings using a P7 HI model with temperature modulation during an HI insult, are consistent with this evidence. Here we find beneficial effects of lower temperatures on certain behavioral tasks. Specifically, hypothermia clearly benefitted HI females on a rota-rod task (HI hypothermic better than HI normothermic), and also on MWM and NSM. Here, normothermic HI females were significantly worse than shams, but HI hypothermic females were not. No benefit was seen for HI hypothermic females on the SG 0–100 task (significantly worse than shams). For males, some benefit was seen on SG 0–100, since HI normothermic (but not HI hypothermic) were significantly worse than sham males. A similar benefit was seen for males on NSM, but no benefit was seen for MWM (HI hypothermic males were significantly worse than sham males on this task). Clearly, male and female P7 HI animals responded differently to temperature modulation, with females benefitting slightly more (overall) than males (3 out of 4 tasks *versus* 2 out of 4 tasks showing protection). However, it is unclear what underlying mechanisms underlie this sex-specific interaction between HI injury, sex, and temperature. We intend to complete a follow-up study investigating histological measures that might be associated with the sex and task-specific beneficial outcomes seen in the current dataset. We will be investigating volumes of the cortex, hippocampus, lateral ventricles, internal capsule, and corpus callosum to determine if specific brain areas are vulnerable to the protective effect of hypothermia. In fact, in related rodent studies, it has been suggested that lower temperatures suppress caspase-3 activity in the cell death cascade [74], which might be related to increased damage in certain brain areas. This is particularly interesting, since evidence shows that females preferentially activate the caspase-dependent cell death pathway, in which caspase-3 is highly implicated [76]—possibly explaining the slight behavioral advantage we see in females in response to lower temperatures. Other animal studies investigating sex differences in the effects of hypothermia-inducing drugs (*i.e.*, cholinesterase inhibitors such as rivastigmine) indicate that testosterone might suppress their temperature lowering effects [92]. This could relate to the current results, since P7 male animals have more circulating testosterone than P7 females [93], and thus testosterone could be blocking some of the protective benefits of hypothermia in male animals.

## 5. Conclusions

It is clear from the results of the current study that temperature modulation during and immediately after an HI insult is important for beneficial results. In animal studies, it is crucial to regulate temperature during experimentally induced HI in order to yield valid outcome data. To our knowledge, this is one of the few known studies examining the beneficial effect of lower temperatures during an HI insult in P7 HI male and female rats and the current study yields important and exciting results. Specifically, the beneficial effect of cooling in male and female HI animals seems to be “task specific,” which suggests that lower temperatures might affect specific brain mechanisms differently. For example, the brain areas responsible for auditory processing, learning/memory, and motor ability may display differential vulnerability to the neuroprotective effect of lower body temperatures in males and females. This could account for differences seen for each sex on each behavioral task. Future research on sex differences, HI, and cooling should also investigate anatomical measures associated with the aforementioned behavioral processes (e.g., MGN, hippocampus, and basal ganglia) to possibly pinpoint areas that benefit the most from lower body temperatures. Additionally, white matter areas such as the corpus callosum and internal capsule should also be examined, since cooling is known to have a specific effect on white matter [94,95]. Finally, further cellular measures should also be employed to examine whether cellular connectivity is affected by cooling in either male or female HI injured animals. In fact, this might be a more likely explanation, since we have shown that male and female HI injured rats display different behavioral outcomes despite similar degrees of brain damage—Implying that cellular-level plasticity is likely to underlie behavioral protection [51]. Overall, the current findings are consistent with the well-established benefits of hypothermia as a neuroprotectant in the clinical HI population. In addition, our results highlight the importance of employing both males and females in pre-clinical and clinical research. Finally, our results suggest a possible need to investigate sex-specific neuroprotection, so that male and female neonates can receive optimized interventions to reduce brain damage and behavioral deficits stemming from an HI insult.
